# The Role of Private Health Sector for Tuberculosis Control in Debre Markos Town, Northwest Ethiopia

**DOI:** 10.1155/2018/8697470

**Published:** 2018-01-28

**Authors:** Alemayehu Reta, Addis Simachew

**Affiliations:** ^1^Department of Medical Laboratory Science, College of Health Sciences, Debre Markos University, Debre Markos, Ethiopia; ^2^School of Medicine, Debre Markos University, Debre Markos, Ethiopia

## Abstract

**Background:**

Tuberculosis has been declared to be a global epidemic. Despite all the effort, only less than half the annual estimated cases are reported by health authorities to the WHO. This could be due to poor reporting from the private sector. In Ethiopia, tuberculosis has also been a major public health problem. The aim of this study was to assess the role of the private health sector in tuberculosis control in Debre Markos.

**Methods:**

An institution based cross-sectional descriptive study was carried out in private health facilities. A total of 260 tuberculosis suspects attending the private clinics were interviewed. Focus group discussion, checklist, and structured questionnaire were used.

**Results:**

Majority of the private clinics were less equipped, poorly regulated, and owned by health workers who were self-employed on a part-time basis. Provider delay of 4 and more months was significantly associated higher likelihood of turning to a private provider (OR = 2.70, 95% CI = (1.20, 6.08)).

**Conclusions and Recommendations:**

There is significant delay among tuberculosis patients. Moreover, there is poor regulation of the private health sector by public health authorities. The involvement of the private sector in tuberculosis control should be limited to identification and refer to tuberculosis cases and suspects.

## 1. Introduction

Tuberculosis remains one of the top 10 causes of death worldwide in 2015. The global plan, to achieve The End TB Strategy by 2020, set 4-5% per year decline rate of tuberculosis (TB) incidence, but 2015 report shows 1.5% between 2014 and 2015 [1]. In 2015, 6.1 million new TB cases were reported to World Health Organization (WHO) by national authorities [2].

Tuberculosis has been considered a major public health problem as far back as 40 years ago and still remains so in Ethiopia, and a functional effort to control tuberculosis was started in the early 1990s by opening the central office of the national TB control program. The program gives free access to TB screening and anti-TB drugs, which were not provided in private sector [3].

According to WHO 2016 report estimates, Ethiopia stands 7th in the list of high burden countries for TB [2, 3].

Most of the few available health institutions are underequipped, and there is generally an insufficient spending on cost-effective health care. Failure by the underresourced public health care services to fulfill their responsibility to deliver health care to all parts of the population led to passive privatization [3–5].

If the private health sector persists and grows as an alternative and unregulated source of care, National Tuberculosis Program (NTP) will be hampered in reaching their case detection targets. The epidemiological impact of the DOTS programs could be diluted in the private health sector by poor case management practice. Such practices, if unchecked, could contribute to the evolution and spread of multidrug-resistant tuberculosis (MDR-TB) [6].

Studies which are conducted in India [7], Vietnam [8], Kenya [9], Kampala [10], and Ethiopia [11] revealed that most of the patients visit private health institutions which still having poor health care facilities and tuberculosis management.

In the context of increasing privatization of health care, countries should introduce innovative methods for optimal cooperation and coordination between the two sectors to improve the quality of health care delivery in general and for the betterment of tuberculosis control in particular [12], and this study will be an additional input.

## 2. Materials and Methods

Institutional based cross-sectional study was conducted to assess the role of private health sectors in TB control from February to March 2017. Around 378 participants (all TB cases or suspects who visited private health centers) were selected by using purposive sampling technique [13].

### 2.1. Data Collection and Analysis

First structured questionnaire and checklists were designed (available here) and then training was given to data collectors for the purpose of study and procedures of data collection for two days prior to the study. After completing the training, a pretest was conducted at the nonstudy subject in a similar population. Problems detected with language, comprehension, instructions, an inadequacy of content, and ease of answering the question were corrected before the questionnaire was finally adopted. Data were collected by face to face interviewing of the study subjects using structured questionnaire and checklists. Qualitative data were collected by employing focus group discussion method with patients attending the Directly Observed Treatment (DOT) centers. The quality of data was controlled starting from the time of questionnaire preparation. For data processing and analysis, SPSS version 20 and EPI data were used. Data were checked for completeness and consistency, and coded data were entered into computer programs after the required cleaning. Logistic regression analysis was used to control confounding effects of multiple exposure variables on the dependent variable. Chi-square test, OR, and 95% CI were used to detect statistically significant differences and associations, *p* values < 0.05 were considered significant.

### 2.2. Ethical Consideration

An ethical clearance was obtained from public health department; also informed consent was obtained from each study subject after the purpose of the study was explained. Confidentiality of the information was assured, and the privacy of the respondents was maintained and could refuse at any stage of the study.

## 3. Result

The majority of the subjects (61.9%) visited public health facilities while 34.6% visited private clinics and pharmacies (private providers) (Table 1).

### 3.1. Associated Factors

There was no statistically significant association between sex, ethnicity, religion, marital status, and residence of the study subjects and the type of provider visited.

In the univariate analysis, age less than 45 years, family size less than five, being literate, and being merchant were significantly associated with higher likelihood of turning to a private provider. However, these associations were nullified in the logistic regression.

Average monthly income of more than 500 Birr and being student were significantly associated with higher likelihood of turning to a private provider both in the univariate analysis and in the logistic regression model (adjusted OR = 2.55, 95% CI = 1.42, 4.57 and Adjusted OR = 2.54, 95% CI = 1.11, 5.82, resp.) (Table 2).

### 3.2. Screening for Tuberculosis

Out of the 260 study subjects, 40% were not screened for tuberculosis on their visit to private clinics or public health facility who were supposed to be screened. There was no statistically significant association between sex, residence, ethnicity, occupation, duration of illness of the study subjects, and being screened for tuberculosis.

Age less than 45 years, being orthodox, and being literate were found to be positively associated with not being screened in the univariate analysis. Nevertheless, these associations were not significant in the binary logistic regression model. Turning to a private provider was found to be positively associated with not being screened for tuberculosis (Adjusted OR = 1.84, 95% CI = 1.21, 2.78). On the other hand, compared to those with income of 500 Birr and more, those with income of less than 500 Birr were more likely to be screened (Table 3).

### 3.3. Health-Seeking Behavior

It is shown that there were no statistically significant associations between duration of illness, traveling time, patient satisfaction, and the type of provider opted for by the study subjects.

There was a significant patient delay with 50.1% of the patients having a delay of four or more months before their first contact with a provider. The mean patient delay was 5.3 months with the standard deviation of 5.6 months, while the median patient delay was four months. But when it comes to provider delay, the majority (78%) was delayed for less than 4 months before starting anti-TB chemotherapy. The mean provider delay was 2.3 months with the standard deviation of 4.9 months. It was found that provider delay of more than four months was strongly associated with turning to the private provider on the first visit among tuberculosis patients (OR = 2.61, 95% CI = (1.17, 15.55)).

On the other hand, there was no statistically significant association between duration of illness before the first visit (patient delay) and the type of provider opted.

Paying service charge of 100.00 Birr and more was significantly associated with higher likelihood of a visit to a private provider both in the univariate analysis and in the logistic regression model (OR = 2.70, 95% CI = 1.20, 6.08).

Likewise, waiting time of three hours and less was significantly associated with higher likelihood of a visit to a private provider with increasing association as waiting for time decreases (OR = 2.67, 95% CI = (1.79, 6.16)) (Table 4).

### 3.4. Private Clinics (Staffing and Practice)

For the second part of the quantitative study, the necessary data were collected from 13 out of 14 clinics in Debre Markos Town; only one of the owner clinics refused to participate in the study. Private clinics in Debre Markos were staffed by high-level professionals like eight specialists, nine general practitioners, and 14 laboratory technicians to list some. Surprisingly, minority of the respondents responded that, tuberculosis can be treated in the private sector, and only 2 out of 13 said that they want to treat TB patients in their clinics. The majority responded that tuberculosis treatment is affordable for few patients. TB control program manual is available only in 4 out of 13 clinics, and only one clinic had a separate register for tuberculosis patients. Only 3 out of 13 (23%) of the clinics had the necessary reagents and facility for AFB microscopy.

All the respondents agreed that both sectors could collaborate in tuberculosis control. When it comes to possible areas for collaboration between the two sectors, 53.8% responded that the collaboration should be in multiple areas like diagnosis and treatment of tuberculosis, patient referral, reporting, and common training and workshops (Table 5).

### 3.5. Summary of the Focus Group Discussion

There was one group consisted of eight participants. Participants were selected with purposive sampling to include opinionated and articulate individuals.

Qualitative data were elaborated and summarized immediately after the sessions by the moderator.

The following principal topics were covered in these focus groups.

#### 3.5.1. Problems Faced by Patients in the Private and Public Sectors

Absence of bacteriological follow-up, lack of trained staff, high cost of drugs, lack of diagnostic facilities, inadequate health education, and lack of mechanisms for defaulter tracing were mentioned as the main problems faced by tuberculosis patients in the private for-profit sector. It was said that these problems could lead to a delay in diagnosis. On the other hand, overcrowding, long waiting time, lack of confidentiality, poor patient-doctor relationship, and poor handling of patients sent from the private sector including requesting reexamination of the sputum of patients who had their sputum already examined in the private sector were mentioned as some of the problems in the public sector. It was said that these problems could lead to a delay of diagnosis or default from diagnostic process especially for patients from rural area due to high cost of accommodation.

#### 3.5.2. Requirements for Tuberculosis Control That Can Be Fulfilled by the Private Health Providers

The private health providers can actively participate mainly in screening TB suspects, reporting, and referral of tuberculosis patients for treatment to public facility. The discussants stressed that it is very difficult to provide anti-TB drugs to the private providers due to difficulty of monitoring them. Moreover, it was indicated that the private sector may not be well motivated to supervise treatment and follow patients throughout the long treatment period; instead it would be more interested in new patients who would be able and willing to pay. Therefore, it was said that, unless there is strong monitoring system in place, it is better to limit the responsibility of the private clinics to diagnosis and referral of patients.

#### 3.5.3. Possibilities and Areas of Collaboration

The participants stated that there should be better communication and collaboration between private and public facilities than the prevailing condition. Diagnosis and referral of patients and using available diagnostic facilities in common were mentioned as some of the important areas for collaboration between the two sectors. It was said that the government should create conducive environment for private sector development based on legal framework and enforce both the private and the public sectors to comply with state-of-art tuberculosis control strategy by regular supervision, monitoring, regulation, and capacity building. They also stressed that the public sector should build its own capacity to be able to take the ultimate responsibility of monitoring and supervising itself and the private sector.

#### 3.5.4. Quality of Health Service Being Delivered

They indicated that there is a better opportunity of getting treatment for the poor in the public sector than that in private sector, though they also indicated that it is more difficult for the poor with the increasing hierarchy in the public health facilities. For instance, it was stated that there was better equity in Debre Markos Health Center than that in DMRH. The participants mentioned that the quality of patient doctor relationship was better in the private sector than that in public facilities. Confidentiality and privacy of patients were said to be well guaranteed by the private providers compared to the public providers. Nevertheless, the discussants stated that they doubt if the private sector was providing appropriate and quality drugs.

## 4. Discussion

This study revealed that a considerable proportion (34.6%) of the patients with TB or symptoms of TB in Debre Markos town had been in contact with a private health care provider either for screening or for anti-TB treatment initiation. There was also similar finding from a study conducted in Bahir Dar on health-seeking behavior of TB patients, which reported that 32.4% of TB suspects turned to the private sector during their first visit [14]. However, in the study conducted in Bahir Dar, unlike in the present study, significantly higher proportion of patients (12.9%) visited traditional healers, while in this study only 0.7% reported a visit to traditional healers. The possible explanation could be the difference in the study population. In the present study, the cases were new patients who were about to start anti-TB or on the investigation while the patients in the Bahir Dar study were already on treatment. In such a situation, the subjects in the present study would be more reluctant to reveal visit to traditional healers since they were on the way to be treated in the modern health care which could lead to more social desirability bias compared to those patients in Bahir Dar who were already on treatment who might be more willing to reveal their behavior long before starting their treatment.

There was no statistically significant association between different sociodemographic factors and visit to the private provider except income and being student. The positive association between higher income and higher likelihood of a visit to the private provider in this study could be due to the fact that the service charge in the private sector was higher than that of the public sector. Hence, this difference in the service charge could be one of the reasons why people with higher income opted for the private sector possibly for better courtesy, less waiting time, and privacy as it was indicated in focus group discussions.

Early detection of TB patients by sputum smear microscopy among symptomatic patients reporting to health services is one of the strategies in TB control. The policy is that all patients with cough of more than two weeks are supposed to give two consecutive sputum samples to be the screen for possible tuberculosis [3]. In this study, there is a significant positive association between a visit to private sector and not being screened. This could be explained by the fact that only three out of the thirteen private clinics, which had the necessary facility for AFB microscopy, might have contributed to less or delayed screening in the private sector.

Having a waiting time of three hours and less is also positively associated with visit to private provider, with higher strength of association with decreasing waiting time (Table 4). Less overcrowding of patients in the private sector compared to the public sector could be one of the explanations for this association.

The positive association between not knowing someone with tuberculosis and higher likelihood of a visit to a private provider (OR = 1.96, 95% CI = 1.15, 3.35) could be due to the fact that those who do not know someone with TB would be less likely to know the TB program and the availability of free treatment and might still opt for the private provider. On the other hand, those who knew someone with TB would be more informed about the TB program, especially taking into consideration the fact that the overwhelming majority of them responded that they knew someone from the family member. This might create more awareness for the patient and the patient's family about the presence of the program in the public sector and might lead them to opt for the public provider.

The delay between the onset of tuberculosis symptoms, diagnosis, and initiation of anti-TB chemotherapy is an important factor in tuberculosis control program. Especially, due emphasis should be given to smear-positive patients delay since they are the most infectious.

A study done in Sidama on delay in treatment of pulmonary tuberculosis revealed that patients with longer duration of illness had a greater number of bacilli on direct microscopy in their sputum. This might lead to increased excretion of bacilli from these patients, which would further aggravate transmission. One of the important strategies in tuberculosis control is early detection of smear-positive tuberculosis cases and treating them with an appropriate combination of chemotherapy to render them noninfectious [3].

Two types of delays were assessed in this study. The first type of delay was a patient delay.

This was defined by the duration of illness from the date of onset till the first visit to a health facility.

Delay of any type should be seriously seen among smear-positive pulmonary TB cases due to its public health impact resulting from its infectiousness. Among smear-positive TB patients starting anti-TB treatment, mean patient delay was 5.3 months with a standard deviation of 5.6 months. This is significantly longer than the mean patient delays reported by other studies. For instance, Lonnroth et al. reported the mean patient delay of 3.1 weeks among smear-positive patients in Ho Chi Minih City, Vietnam [8]. There was also significant provider delay. It was found that provider delay of more than four months was strongly associated with turning to a private provider on the first visit among tuberculosis patients (OR = 2.61, 95% CI = (1.17, 15.55)). The possible explanation for this association could be the delay or absence of screening in the private clinics due to lack of screening facilities which would increase the provider delay by delaying the diagnosis of the patient.

## 5. Conclusions and Recommendations

Considerable numbers of tuberculosis patients visit the private health sector. There is significant provider delay before initiating treatment for TB patients especially for those patients who first visited the private provider. Underscreening of tuberculosis suspects that is more significant in the private sector compared to the public sector. There is a lack of appropriate coordination and collaboration between the private and public sectors in tuberculosis control. The majority of the private clinics in Debre Markos are unregulated and unsupervised and lack the necessary requirements for effective tuberculosis control like sputum microscopy, NTP manual, TB register, trained staff, and regular working hour.

There is a need to establish better communication, coordination, and referral of patients between private and public sectors and also to monitor, supervise, and regulate the private health sector, in order to achieve the objectives of tuberculosis control program in the study area. The health workers in the private sector should be trained to be able to early screen or refer tuberculosis patients in case there is no screening facility in the private sector. To reduce repeating sputum examination for patients who had their sputum did in the private sector, the tuberculosis control program should better establish quality control and close supervision to the private clinics to ensure the validity of the result from the private sector. Health education about tuberculosis should be conveyed to patients and the communities by different media like radio, billboards, and brochures to create awareness about tuberculosis so that they report early when they have symptoms of tuberculosis. Operational research in possible involvement of the private sector in service delivery in TB control (like case detection, diagnosis, and treatment delivery) in the study area should be conducted.

## Figures and Tables

**Table 1:** Frequency of visit by patients with symptoms of TB to different facilities, Debre Markos Town, February to March 2009.

Characteristic	Frequency	%
Public facility	161	61.9
Private clinics	78	30
Private pharmacies	12	4.6
Traditional	1	0.3
Wholly water	8	3

**Table 2:** Sociodemographic characteristics of study subjects versus provider visited first in Debre Markos Town, February to March 2009.

Characteristics	Private, *N* = 90 (%)	Public, *N* = 161 (%)	Crude OR (95% CI)	Adjusted OR (95% CI)
Age (years)				
< 45	76 (84.4)	128 (79.5)	1.42 (0.77, 2.66)	—
>45	14 (15.6)	33 (20.4)	1^∗^	—
Sex				
Male	48 (54.6)	85 (52.3)	1.10 (0.80, 1.51)	—
Female	42 (45.4)	76(47.7)	1^∗^	—
Address				
Urban	31 (70.4)	52 (64.6)	1.27 (0.90, 1.79)	—
Rural	14 (29.6)	29 (35.4)	1^∗^	—
Family size				
< 5	54 (60.0)	92 (56.7)	1.17 (0.82–1.68)	—
>5	36 (40.0)	69 (43.3)	1^∗^	—
Occupation				
Farmer	26 (28.8)	52 (32.2)	1^∗^	1^∗^
Housewife	21 (23.3)	41 (25.4)	1.19 (0.60, 2.36)	1.10 (0.55, 2.21)
Unemployed	12 (13.3)	27 (16.6)	1.60 (0.73, 3.55)	1.10 (0.45, 2.27)
Student	13 (14.4)	15 (9.3)	2.38 (1.14, 4.96)	2.54 (1.11, 5.82)^a^
Employee	8 (8.8)	16 (9.9)	2.52 (1.12, 5.69)	1.47 (0.63, 3.42)
Merchant	12 (13.3)	10 (6.2)	1.26 (0.68, 2.35)	1.08 (0.54, 2.17)
Literacy				
Literate	34 (37.3)	46 (29.6)	1.45 (1.04, 2.03)	1.04 (0.65, 1.70)
Illiterate	56 (62.7)	115 (71.4)	1^∗^	1^∗^
Average monthly income				
(ETB) < 500	74 (82.2)	147 (91.3)	1^∗^	1^∗^
>500	16 (17.8)	14 (8.7)	2.56 (1.56, 4.22)	2.55 (1.42, 4.57)^a^

^a^Statistically significant association; ^∗^referent values.

**Table 3:** Comparison of screened and nonscreened TB suspects by their demographic and health-seeking characteristics, Debre Markos Town, February to March 2009.

Characteristics	Nonscreened (*n *= 104) (%)	Screened (*n *= 186) (%)	Crude OR 95% CI	Adjusted OR 95% CI
Age (years)				
< 45	85 (85.5)	127 (78.2)	1.63 (1.05, 2.52)	—
>45	19 (14.6)	29 (21.7)	1^∗^	—
Sex				
Male	56 (55.5)	85 (52.3)	1.07 (0.78, 1.48) 1^∗^	—
Female	45 (45.2)	74 (47.7)		—
Address				
Urban	71 (68.3)	101 (64.6)	1.00 (0.71, 1.40)	—
Rural	33 (31.7)	55 (35.4)	1^∗^	—
Family size				
< 5	20 (19.3)	37 (23.7)	0.998 (0.24, 1.24)	—
>5	84 (80.7)	119 (63.3)	1^∗^	—
Occupation				
Farmer	32 (30.7)	49 (31)	1.33 (0.72, 2.47)	—
Housewife	25 (24.0)	38 (24.3)	0.99 (0.49, 1.98)	—
Unemployed	18 (14.4)	23 (16.7)	2.04 (0.97, 4.29)	—
Student	11 (11.5)	18 (11.5)	1.16 (0.50, 2.66)	—
Employee	10 (9.6)	14 (8.9)	1.24 (0.53, 2.87)	—
Merchant	8 (7.6)	14 (8.9)	1^∗^	—
Literacy				
Literate	38 (36.5)	44 (28.2)	1.46 (1.04, 1.07)	—
Illiterate	66 (63.5)	112 (71.8)	1^∗^	—
Average monthly income				
< 500	67 (65.0)	121 (77.8)	0.53 (0.37, 0.76)	0.55 (0.34, 0.89)^a^
> 500	37 (35.0)	35 (22.2)	1^∗^	1^∗^
Private	58 (55.8)	32 (19.1)	5.68 (3.94, 8.20)	1.84 (1.21, 2.78)^a^
Public	46 (44.2)	154 (88.4)	1^∗^	1^∗^

^a^Statistically significant association; ^∗^referent values.

**Table 4:** Health-seeking behavior and other characteristics of patients with symptoms of tuberculosis by provider visited first, Debre Markos Town, February-March 2009.

Characteristics	Private *N* = 90 (%)	Public *N* = 161 (%)	Crude OR 95% CI	Adjusted OR 95% CI
Duration of illness before 1st visit				
< 4 months	46 (51.1)	79 (49.1)	0.99 (0.60, 1.65)	—
>4 months	44 (48.9)	82 (50.9)	1^∗^	—
Provider delay				
< 4 months	60 (66.6)	136 (84.6)	1^∗^	1^∗^
>4 months	30 (33.4)	25 (15.4)	5.38 (1.17, 28.03)	10.44 (1.17,93.36)^a^
Travelling time				
< 3 hrs	76 (84.4)	138 (85.2)	0.67 (0.31, 1.47)	—
>3 hrs	14 (14.6)	23 (14.8)	1^∗^	—
Service charge				
< 100.00	20 (22.2)	132 (81.4)	1^∗^	1^∗^
>100.00	70 (77.8)	29 (18.6)	3.75 (1.73, 8.33)	2.70 (1.20, 6.08)^a^
Difficulty to pay for service				
Difficult	60 (66.7)	90 (55.6)	1.60 (1.2, 2.28)	—
Not difficult	30 (33.3)	71 (44.4)	1^∗^	—
Satisfaction with time given				
Satisfied	80 (89.4)	142 (88.5)	1.09 (0.63–1.91)	—
Not satisfied	10 (10.6)	19 (11.5)	1^∗^	—
Satisfaction with cost				
Satisfied	74 (83.8)	142 (88.1)	0.70 (0.43, 1.14)	—
Not satisfied	16 (16.2)	19 (11.9)	1^∗^	—
Overall satisfaction				
Satisfied	82 (91.9)	144 (89.1)	1.39 (0.76–2.60)	—
Not satisfied	8 (8.1)	17 (10.9)	1^∗^	—
Waiting time				
< 3 hrs	86 (95.5)	132 (81.5)	2.65 (1.17, 6.17)	2.67 (1.79, 6.16)^a^
>3 hrs	4 (4.5)	29 (18.5)	1^∗^	1^∗^
Know someone with TB				
No	66 (75.3)	120 (72.7)	1.14 (0.77, 1.69)	1.96 (1.15, 3.35)^a^
Yes	24 (24.7)	43 (27.3)	1^∗^	1^∗^

^∗^Referent values; ^a^statistically significant associations.

**Table 5:** Observations and responses by private provider concerning tuberculosis control, Debre Markos Town, February to March 2009.

Characteristics	Frequency	(%)
Measures taken for TB suspects diagnosed in the clinic		
Referral to public facility	1	7.7
Diagnosed and referred to public facility	6	46.2
Appointed for reevaluation	5	38.5
Collaborate in TB control	1	7.7
Yes	13	100
No	0	0
Can TB be treated in the private clinic		
Yes	3	23.1
No	10	76.9
Do you want to treat TB in your clinic		
Yes	2	15.4
No	11	84.6
Affordability of anti-TB drugs		
Some can afford	2	15.4
Few can afford	7	53.8
None can afford	2	15.4
Do not know	2	15.4
Availability of TB registers		
Available	1	7.7
Not available	12	92.3
Availability of a TB control program manual		
Yes	4	30.8
No	9	69.2
Availability of a TB control program manual		
Yes	4	30.8
No	9	69.2
Availability of trained staff		
Available	6	46.2
Not available	7	53.8
Do you educate your patients about TB		
Yes	10	76.9
No	3	23.1
Visit from government in the last three months		
Yes	3	23.1
No	10	76.9
Reagents for AFB		
Available	3	23.1
Not available	10	76.9

## Supplementary Materials

Supplementary Materials Based on the reviewer's recommendation, we try to incorporate the questionnaires for TB suspects or patients attending private clinics during the study period either for screening or treatment initiation. It contains variables like socioeconomic factors, type of health facility, duration of illness, clinical features, treatment protocol, and patient satisfaction.
