# Supplementary material for: The Role of Private Health Sector for Tuberculosis Control in Debre Markos Town, Northwest Ethiopia
Source: Adv Med. 2018 Jan 28;2018:8697470. doi: 10.1155/2018/8697470 (PMC5896415; doi:10.1155/2018/8697470)
Supplement: Supplementary Materials — Based on the reviewer's recommendation, we try to incorporate the questionnaires for TB suspects or patients attending private clinics during the study period either for screening or treatment initiation. It contains variables like socioeconomic factors, type of health facility, duration of illness, clinical features, treatment protocol, and patient satisfaction. [file 8697470.f1.docx]

Questionnaire for TB suspects or patients attending private clinics during the study period either for screening or treatment initiation.

| No. | Questions | Answers |
| --- | --- | --- |
| 001 | Card no |  |
| 002 | Sex | 1.Male  2.Female |
| 003 | How old are you? | Years………….. |
| 004 | Address | …………….. |
| 005 | What is your religion? | 1.Orthodox  2. Muslim  3. Protestant  4.Catholic  5.Other |
| 006 | What is your ethnicity? | 1.Amhara  2.Oromo  3.Tigrawi  4.Other |
| 007 | What is your marital status? | 1.Married  2.Single  3.Widowed  4.Divorced  5.Separated |
| 008 | Educational status | 1. Can read and write  2.Can write  3. Can read  4. Cannot read and write |
| 009 | What is your occupation? | 1. Farmer  2. Merchant  3.Employed(Gov. /private)  4.Unemployee  5.House wife  6. Student |
| 010 | What is your family size?(children, spouse, including other family members) | …………….. |
| 011 | What is your family’s average monthly income? | ………… |
| 12 | For how long you have been ill  Since the onset of symptoms to your first contact to any health care provider? | ……………. |
| 013 | For how long have you been ill  From your first contact to any health care provider till you sought help (diagnosis)? | …………….. |
| 014 | Which health facility did you visit first for your current illness episode? | 1. Private pharmacy  2. Private clinic  3. Government hospital  4. Government health center  5. Government health post  6. Traditional healer  7.Wholly water  8. Other (specify)…………………. |
| 015 | In relation to question No.016, Why did you choose the place you mentioned above to visit first? | 1. Nearest place to home  2.Good reputation(name)  3.Pleasant reception  4.Cheaper price  5.Other (specify) |
| 016 | What was done for you at the health facility you visited first for your current illness? | 1.Sent me for sputum examination  2.Sent me to start treatment for TB  3.He/she prescribing me medication  4. Other (specify)………………………… |
| 017 | Status of the patient? | 1.Sputum examined positive for TB  2.Sputum examined negative for TB  3.Others(specify) |
| 018 | Where did you have your sputum  Examined? | 1.Private clinic  2.public health facility  3.No where |
| 019 | How did you come to this clinic? | 1. Sent from private clinic  2.Sent from government facility  3. Personal decision  4.Advise from relatives and friends |
| 020 | How did you come from your residence to this center? | 1.On foot  2.By taxi  3.On back of animals  4.By bus  5. Other (specify)………………………… |
| 021 | Do you know your current illness? | 1. Yes (specify)………….  2.No |
| 022 | If no. to 021/ what do you think it  could be? | Specify………………. |
| 023 | Do you know somebody sick with tuberculosis? | 1.Yes, a member of my family  2.Yes, my friend  3.Yes, my neighbor  5.No |
| 024 | Can you come daily to this center for treatment at least for two months? | 1.Yes  2.No |
| Now I am going to ask you about the health provider and the care you received in the  facility you visited either for diagnosis or treatment (remember the answerQ014) | | |
| 025 | How long did you wait before seeing adoctor? | 1.Minutes  2.Hours  3.Days |
| 026 | How satisfied are you with the time the health worker spent with you during your visit? | 1.Very satisfied  2.Satisfied  3.Dissatisfied  4.Very dissatisfied |
| 027 | How much did you pay for the service? | ……………. |
| 028 | Who paid the cost? | 1.Self (within family)  2. Insurance  3.Exempted  4.Other |
| 029 | How difficult was finding the money to pay for the health care ? | 1.not difficult  2.Difficult  3.Very difficult |
| 030 | Do you satisfied by the level of service charge you ? | 1.Very satisfied  2.Satisfied  3.Dissatisfied  4.Very dissatisfied |
| 031 | How satisfied are you in overall with the services you received from the health worker? | 1. Very satisfied  2. Satisfied  3.Dissatisfied  4.Very dissatisfied |

Checklist for private health providers Debremarkos Town, 2009.

| 001 | The level of the clinic | 1. Higher clinic  2. Medium clinic  3.Small clinic |
| --- | --- | --- |
| 002 | Is the respondent the owner of the institute? | 1.Yes  2. No |
| 003 | What is the qualification of the owner of the institute ? | ……………………………… |
| 004 | Type and number of professionals in the institute  Specialist………………………  General practitioner..................  Health officer………. Nurse…….................  Lab technicians…………………  Pharmacist ………………………  Pharmacy technician……………  Druggist………………………  X-ray technician………………  Other………………………… | Full time Part-time  ……….. ………..  ………… ……….  ………… ……..  ………… ……….  ………… ……..  ………… …….  ………… ………  ………… ……..  ………… ………….  ………… ……….. |
| 005 | Is there reagents for AFB staining are available? | 1. Yes  2 .No |
| 006 | Is there any other diagnostic facility for tuberculosis in this clinic ? | (specify)…………….. |
| 007 | What measures were taken for tuberculosis suspects? | 1.Diagnosed in the clinic  2.Given appointment for re-evaluation  3.Referred to other private clinic  4. Referred to public facility  5.appointed for reevaluation |
| 008 | What tool used to diagnose theTB cases? | History and physical examination...............1  Sputum for AFB……2  X-ray……………….3  Other (specify)……………………… |
| 009 | Is there any TB case on treatment in the clinic? | 1.Yes  2.No |
| 010 | Is there a registration book for TB patients on treatment? | 1.Yes  2.No |
| 011 | Is there a national Tuberculosis control program manual in the clinic? | 1.Yes  2. No |
| 012 | Do you want to treat TB patients in your clinic? | 1.Yes  2. No |
| 013 | Are anti-TB drugs available in your clinic? | 1.Yes  2. No |
| 014 | Do you think that tuberculosis can be treated in the private clinic? | 1.Yes  2. No |
| 015 | Can the patients afford ant-TB treatment? | 1. Yes most can afford  2.Yes some can afford  3.Yes few can afford  4.No one can afford  5. I Don't know. |
| 016 | Do you have somebody trained in tuberculosis control among  Your staff in the last one year? | 1.Yes  2.No |
| 017 | Do you teach TB patients who attend your clinic about their illness? | 1. Yes (specify some contents)…………………….    2.No |
| 018 | Is it possible for the private and government sectors to collaborate in tuberculosis control? | 1.Yes  2.No |
| 019 | If yes for Q 18 Collaboration should be in: | 1.Diagnosis of TB  2.treatment  3. Patient referral  4. Designing strategy to control TB  5.Common training and workshops |
| 020 | Do you agree with the DOTS strategy used by the National TB Program to control tuberculosis? | 1.Yes  2.No |
| 021 | Did you have regulatory /supervisory visit from the health authorities in the last three months? | 1.Yes  2.No |
| 022 | Have you a reporting mechanism to health authorities in TB control? |  |
